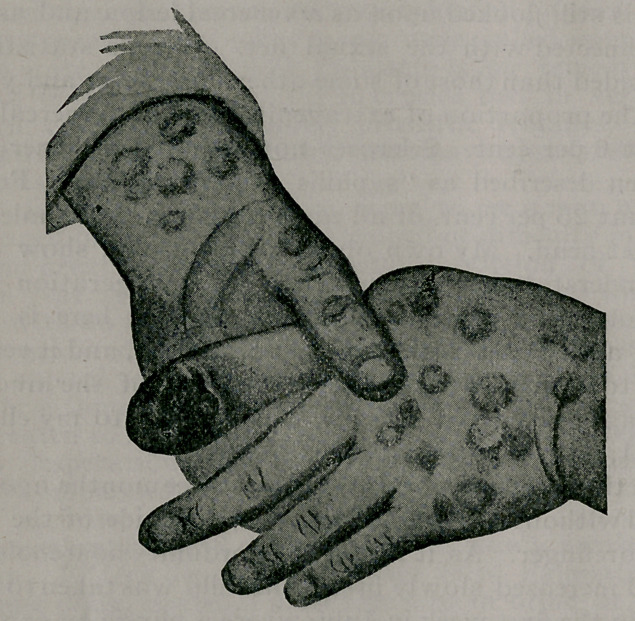# A Rare Syphilitic Infection

**Published:** 1896-02

**Authors:** William S. Gottheil

**Affiliations:** New York, Professor of Dermatology, New York School of Clinical Medicine; Dermatologist to the Lebanon Hospital and the West Side German and the Northwestern Dispensaries; 37 West 50th Street


### A RARE SYPHILITIC INFECTION.

By WILLIAM S. GOTTHEIL, M. D., NEW YORK,

Professor of Dermatology, New York School of Clinical Medicine; Dermatologist to the Lebanon Hospital and the West Side German and the Northwestern Dispensaries.

The chancre, or, as it is better called, the "sclerosis," that marks the point of entrance into the system of the organism that causes the chronic exanthematous disease known as syphilis, is still looked upon as a venereal lesion and as necessarily connected with the sexual act. Julien's statistics are less one-sided than those of some other observers; and yet even he puts the proportion of extragenital and nonvenereal initial lesions at 6 per cent. Scleroses not acquired in venery, have lately been described as "syphilis insontium," and Fournier claims that 25 per cent. of all cases that occur in females come under that head. My own observations would show this to be an understatement rather than an exaggeration of the proportion. The case described and figured here is an example of a rare localization of the initial sore, and it certainly deserves to be classed as a case of "syphilis of the innocent."

A. S , aged seventeen months, was brought to my clinic on October 1, with the following history:

About the beginning of July, some three months ago, a sore appeared without any known cause at the side of the nail of his left forefinger. As it resisted the ordinary household remedies, and increased slowly in size, the child was taken to the dispensary in the first week in July, where a physician cauterized the sore, and prescribed an ointment. By the beginning of August, spots had come on his body, limbs, face and hands. The finger got no better. The child ran down; it became pale and sickly, and "went back on its food." The case was then referred from the surgical to the pædiatric department, and finally came to me.

Examination showed a pale and anæmic boy, with yellow skin and soft, flabby muscles. His entire body, including his face and head, was covered with a closely-sown eruption, consisting of quite large pink papules, in some places topped with a moderate quantity of silvery scales. The palms and soles were markedly affected and here the scales were thicker, and semi-detached at the margins of the papules. Around the anus was a circle of moist papules. A general indolent adenopathy was present. The visible mucosæ were normal; there was no alopecia.

On the baby's left finger was a sore for which the mother first brought him to the institution. The nail was apparently entirely gone, and its place was occupied by a fungating mass of tissue which covered the entire dorsal surface of the distal phalanx. This fungous mass was apparently composed of hypertrophic granulation tissue; and the edges of the skin around it were reddened and indurated. The entire phalanx was as large again as it should be; and the child sought to protect it from injury by holding it with his other hand in a very characteristc manner.

We were evidently dealing with a case of acquired syphilis in its early stages. The initial sclerosis was still present on the forefinger, though evidently retrogressing. It had been there at least twelve weeks; and we know that syphilis resembles its first cousin, the acute exanthemata, in that it goes through its various stages in an orderly manner and with a definite rate of progression whether treated or not. The specific induration was almost gone, and the loss of tissue caused by the breaking down or the interstitial absorption of the syphilitic granuloma was being repaired with granulation tissue.

The general papulo-squamous syphiloderm was florid, and at its height, as was proper at the time. On the hands and face, where soap and water are more freely used, we had the papules alone; over the rest of the body the scales were present in varying, though moderate quantity. The marked general adenopathy only confirmed the visual diagnosis.

Apart from the interest that a case of acquired syphilis in a child so young as this naturally possesses, the question as to the mode of origin of the sclerosis at once presents itself to us. How and where could an infant so young as this acquire a digital chancre? Whilst I cannot answer this question with absolute certainty, I am satisfied that it derived it from its mother.

The child slept with its mother; and sleeping together in the tenement from which this patient came, meant a community of soap, towels, bed-linen and dirt, which rendered the infection of the child, if the mother had the disease, almost a matter of necessity. The mother admitted that she had had sores around the anus in January, which had lasted until August, a month after the child's finger began to get sore. She was sick "all over her body," at the time; but she denies any general eruption or secondary symptoms. She noticed herself that during the time that she was sick her baby lost its power to play and walk, and ran down; for she was suckling it as these people do, until the last possible moment, to avoid pregnancy. But a most careful examination failed to reveal any evidences of present syphilis in her. Her husband, of course, refused to be examined; there is never anything the matter with the husbands!

The subsequent history of the case presents no points of special interest. The baby was given regular mercurial inunctions, the white precipitate ointment being employed for the anal papules. There was steady improvement; the sclerosis slowly retrogressed, and began to heal. The eruption faded. The child began to play and walk again. On November 5, I noted papular eruption hardly visible, finger nearly normal in size, ulceration small. The child being still pale and languid; the syrup of the iodide of iron in five-drop doses three times daily was ordered, the inunctions being still kept up. November 15, the sore on the finger had entirely cicatrized; the nail bed was, however, completely destroyed and a rough and thick scar had taken its place. On March 11, of the following year, when I last saw the child, it looked fat and healthy with a clear complexion and firm musles.

*37 West 50th Street.*

---

THE NECESSITY OF FREQUENT VISITS.—The supreme court of California, says the *Medical Record*, in an action brought by a physician for professional services—the defense being that the visits were too frequent and not necessary—rules that "the defendant having admitted the employment of the plaintiff as a physician to treat his wife and children, the plaintiff was the proper judge of the necessity of frequent visits, and in the absence of proof to the contrary, the court will presume that all the professional visits made were deemed necessary, and were properly made. It would be a dangerous doctrine for the sick to require a physician to be able to prove the necessity of each visit before he can recover for his services. This is necessarily a matter of judgment, and one concerning which no one save the attending physician can decide. It depends not only upon the condition of the patient, but in some degree upon the course of treatment adopted."

## Figures and Tables

**Figure f1:**